# Inter-Vertebral Flexibility of the Ostrich Neck: Implications for Estimating Sauropod Neck Flexibility

**DOI:** 10.1371/journal.pone.0072187

**Published:** 2013-08-14

**Authors:** Matthew J. Cobley, Emily J. Rayfield, Paul M. Barrett

**Affiliations:** 1 School of Earth Sciences, University of Bristol, Bristol, United Kingdom; 2 Department of Earth Sciences, The Natural History Museum, London, United Kingdom; University of Birmingham, United Kingdom

## Abstract

The flexibility and posture of the neck in sauropod dinosaurs has long been contentious. Improved constraints on sauropod neck function will have major implications for what we know of their foraging strategies, ecology and overall biology. Several hypotheses have been proposed, based primarily on osteological data, suggesting different degrees of neck flexibility. This study attempts to assess the effects of reconstructed soft tissues on sauropod neck flexibility through systematic removal of muscle groups and measures of flexibility of the neck in a living analogue, the ostrich (*Struthio camelus*). The possible effect of cartilage on flexibility is also examined, as this was previously overlooked in osteological estimates of sauropod neck function. These comparisons show that soft tissues are likely to have limited the flexibility of the neck beyond the limits suggested by osteology alone. In addition, the inferred presence of cartilage, and varying the inter-vertebral spacing within the synovial capsule, also affect neck flexibility. One hypothesis proposed that flexibility is constrained by requiring a minimum overlap between successive zygapophyses equivalent to 50% of zygapophyseal articular surface length (ONP50). This assumption is tested by comparing the maximum flexibility of the articulated cervical column in ONP50 and the flexibility of the complete neck with all tissues intact. It is found that this model does not adequately convey the pattern of flexibility in the ostrich neck, suggesting that the ONP50 model may not be useful in determining neck function if considered in isolation from myological and other soft tissue data.

## Introduction

The sauropods were the largest terrestrial animals ever to have existed. The clade Sauropoda, a group of saurischian dinosaurs, was immensely successful from the Late Triassic to the very end of the Cretaceous, in terms of both species-richness and numerical abundance, with representatives found on all continents [1], [2]. Whilst their general morphology is well understood, the issue of their neck posture is still contentious. Some recent studies have proposed that the long necks of sauropods evolved by sexual selection [3]; however, the lack of evidence for this theory [4] reinforces the long held view that long necks evolved for maximising the feeding envelope, either for high browsing [5]–[7] or a wider lateral range of low browsing [8]–[10]. Species- or clade-specific variations in neck morphology have also been proposed as the basis for ecologically significant differences in foraging behaviour, mediated by changes in relative and/or absolute neck length as well as differences in neck flexion capabilities [11]. Various theories on the posture and flexibility of the neck have been presented [8], [9], [11]–[15], with differing approaches leading to various implications for overall biology. Whilst heart size and output [16], [17], the structure of the respiratory system [18], [19], risk of predation, and intraspecific niche partitioning [13] are all affected by neck function, there are also major implications for sauropod diet and ecology [11]. Whilst neck posture and flexibility in most species has relatively little effect on their ecology due to their relatively short necks, sauropod necks can reach up to 15 m in length [20], meaning small differences in the angle at which the neck is held can lead to differing head heights of a metre or more. Sauropods display a wide array of body sizes and neck morphologies, but broadly speaking if they were to have a gentle downward curve, the heads of many sauropods would reach heights of 2–4 m, whilst an extreme vertical ‘swan-like’ posture would lead to some species with head heights of 16–20 m [11], [21], [22]. Establishing the flexibility of sauropod necks allows estimation of the ‘feeding envelope’ of a given species. This envelope is the maximum range over which an individual could feed while standing still, and along with previous work on the flora present at the time [23]–[25] and sauropod dentition [11], [13], [26]–[32], allows an inference of possible feeding ecologies. Establishing sauropod diet is extremely important; because sauropods were so large, adults may have required up to 400 kg of dry plant matter per day [24]. Reducing the resources in a given area would force other species present to adapt by either feeding on different material, or through temporal or spatial niche partitioning of the same vegetation [11].

Previous work on sauropod neck posture and flexibility has led to three general theories. Initially, qualitative 2D and 3D comparisons were made on sauropod cervical vertebrae in order to assess the potential osteological limits of neck flexibility (e.g., effects of zygapophyseal overlap, centrum articular surface morphology, cervical rib length, neural spine orientation, etc.). This work suggested varying degrees of dorsoventral and mediolateral flexion in different taxa [8], [9], [11]. A second method introduced computer modelling of the neck [12]. The latter study was the first to propose that vertebrate necks are held in an ‘osteological neutral pose’ (ONP), where two adjacent vertebrae are habitually held with 100% overlap between the pre- and post-zygapophyses. This study also asserted that neck vertebrae could not be flexed beyond a minimum of 50% overlap between the zygapophyses of adjacent vertebrae, a measure referred to hereafter as ONP50 [12]. Application of this method led to low flexibility estimates for sauropod necks, and the conclusion that species such as *Diplodocus* and *Apatosaurus* held their necks in a downward sloping fashion [12], [13], [21], [33]. However, this work was questioned following by a study making direct comparisons between neck postures in extant species: this study hypothesized that as many extant amniotes habitually hold their necks in poses that are flexed dorsally at the cervicodorsal junction, and that this is likely the primitive condition for amniotes [15]. As such it was considered most parsimonious to reconstruct sauropod necks with a more vertical ‘swan-like’, ‘S’-shaped posture [15]. Thirdly, mechanical models have also been implemented, which have supported a middle ground between these two extremes, with the neck being held slightly above horizontal and permitting a reasonable amount of lateral and dorsoventral flexibility [34]–[40]. Finally, other studies that investigated neck flexibility in extant taxa (both on the basis of osteology alone, as well as with soft tissues intact), on *Struthio camelus* (the ostrich), *Giraffa camelopardis* (the giraffe) and *Camelus bactrianus* (the Bactrian camel), have supported these ‘middle ground’ suggestions [14].

None of these previous studies analysed the effects of soft tissue on the flexibility of the neck skeleton: ONP50 relies solely on osteological measurements [12]; the orientation of the neck as a whole has been used as a more superficial means of comparison [15]; and the ‘Preuschoft method’ [39], [40] deals solely in the mechanics of the neck. Studies based on the flexibility of extant animal necks have yet to study the actual effects of soft-tissues and cartilage on the flexibility of the neck, instead comparing the flexibility of the neck with all tissue intact with that of the cervical skeleton. This study aims to rectify this situation. By measuring the flexibility of the neck after sequential and cumulative removal of tissues, a picture of how the soft tissue of the neck affects flexibility becomes apparent. Where previous studies have mainly focused on ONP as a predictor of posture [15], [39], this study analyses ONP50’s suitability as a predictor of estimating maximum flexibility of the neck. The effect of cartilage is also investigated; whilst the presence/absence of the various muscles that control neck movement can be inferred, their masses and origins/insertions within the neck are debateable. The presence of cartilage is to some extent less contentious, yet is something that previous studies have not accounted for. The study was conducted using the ostrich as it has an elongate neck composed of numerous individual segments, as in sauropod dinosaurs. As birds are parts of the dinosaur radiation they also represent part of the extant phylogenetic bracket (EPB) for Sauropoda [41], [42]. Moreover, previous work on ostrich necks facilitates comparisons with previous studies [14], [39]. These analyses are then brought together to evaluate previous methods for estimating posture and flexibility in sauropod dinosaurs.

## Materials and Methods

### Animals Studied

*Struthio camelus* was chosen as an analogue for the sauropod neck using the EPB approach [41], [42]. Sauropods are stem avians, and as the Struthioniformes are the largest birds to exhibit elongate necks, and the overall morphologies of ostrich vertebrae and cervical axial musculature are broadly comparable to those of sauropods (in terms of musculature present, high number of cervical vertebrae, presence of pneumaticity, etc.), ostriches are a suitable candidate for comparative study. However, it should be noted that there are some differences in inter-vertebral articulations between these taxa (heterocoelous in ostriches, opisthocoelous in sauropods) that would affect direct comparisons of their flexibility. It is thought that ratites evolved elongate necks independently on several occasions [43]. Three female ostrich necks were used in this study, donated by MNS Ostriches Ltd, U.K. All three were humanely destroyed at around the same age (∼ 6 months). All three necks had been separated from the torso prior to being obtained; two had been pre-skinned and decapitated, whilst one had its head and skin intact. The necks were frozen immediately after amputation to minimize decomposition, and to reduce the effects of rigor mortis. These specimens are available for examination and other use via contact with the corresponding author.

### Analysis of Flexibility of the Cervical Column

**Figure 1:**
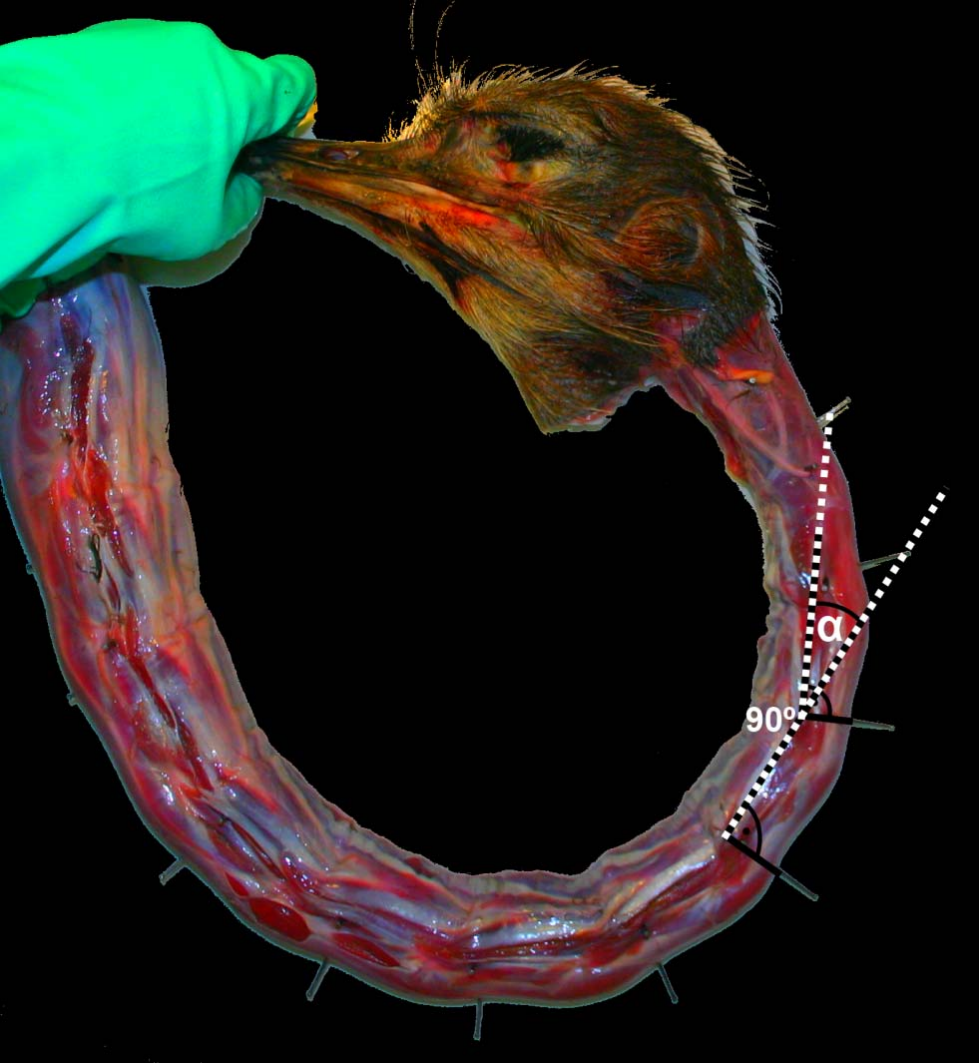
Measuring flexibility in the neck of *Struthio camelus*. Flexibility is measured between adjacent vertebrae as excursions from 0°, where two adjacent centra form a straight line. Dorsal flexion occurs where α >0. Ventral flexion is shown here. Adapted from [14].

The necks were examined immediately after thawing. The flexibility of the neck was measured at various stages of cumulative tissue removal (in sequential order): with all tissue intact; after removal of the long dorsal musculature; after removal of the long ventral musculature; after removal of the lateral musculature; after removal of the single-segment muscles (muscles solely connecting adjacent vertebrae); and after removal of the ligamentum elasticum. These groups are based on the placement of the muscle in relation to the vertebrae rather than their function. The muscles in each group and their attachment sites are detailed in Table 1 (also see: [44]). Flexibility measurements were made using a medical goniometer, measuring the flexibility about each inter-vertebral joint, where flexibility amounted to the degree of movement a given vertebra was capable of in relation to the vertebra immediately posterior (Fig. 1) Flexibility was measured to the nearest half degree. All flexibility measurements are given as deviations from 0°, where the anterior vertebra is angled in a straight line with the posterior vertebra. For the purposes of this paper, we have followed previous studies by representing dorsal excursions as positive, and ventral excursions as negative [14]. Each cervical column was finally cleaned of all soft tissue by being boiled several times in water until all tissue and fat was removed.

**Table 1:** Origins, insertions and groups of the cervical musculature of *Struthio camelus*.

Muscle Group	Muscle	Origin	Insertion
**Dorsal**	**M. biventer cervicis**	Processus spinosus of the posteriorcervical/anterior thoracic vertebrae	Parietals
	**M. longus colli dorsalis**	Processus spinosus	Torus dorsalis
	**M. ascendens cervicalis**	Ansa costotransversaria	Torus dorsalis
**Ventral**	**M. flexor colli medialis**	Processus caroticus	Processus ventralis corporis
		Processus costalis	Processus costalis
	**M. longus colli ventralis**	Processus caroticus	Processus costalis
		Processus ventralis corporis	
**Lateral**	**M. flexor colli lateralis**	Tubercula ansae	Processus costalis
		Cristae laterals	
**Single Segment**	**Mm. intercristales**	Crista transverso-obliqua	Crista transverso-obliqua
	**Mm. insterspinales**	Processus spinosus	Processus spinosus
	**Mm. intertransversarii**	Tubercula ansae	Tubercula ansae
		Cristae laterales	Cristae laterals

Muscles appear in the order removed in this study. Modified from [44].

### ONP50

To test the hypothesis that the flexibility of extant animal necks could be predicted by ONP50 [12], the neck skeleton (with cartilage still present) of the ostrich was oriented to allow a minimum of 50% overlap between the zygapophyses in dorsoventral and lateral movement. This was then compared with the actual values of flexibility allowed by the neck with tissue intact.

### Effects of Cartilage

The maximum degree of flexibility was measured for the ostrich neck skeleton whilst the cartilage was wet (immediately after boiling for 30 minutes to remove the remainder of the soft tissue); after then drying the cartilage; and then again after removal of the cartilage with a scalpel. The ONP50 method was used as a means of evaluating flexibility of the individual joints of a neck skeleton. Rather than to estimate the flexibility of the neck, this was used simply to gauge the effect of cartilage on flexibility. Measurements of neck length along the most dorsal edge of the neck were taken before and after removal of the tissue.

### Naming Conventions Used

Due to the complex nature of the cervical musculature and a previous lack of consensus over the naming of the various muscles, it is important to state the conventions used for naming the various muscles and muscle attachment sites. We follow the nomenclature of the *Nomina Anatomica Avium* [45] herein.

### Other Abbreviations

ONP – osteological neutral pose; ONP50– model that assumes range of motion is restricted to a minimum of 50% overlap of zygapophyseal articular surface length. EPB – Extant Phylogenetic Bracket; C3–C15– cervical vertebrae 3–15.

## Results

### Flexibility

**Figure 2:**
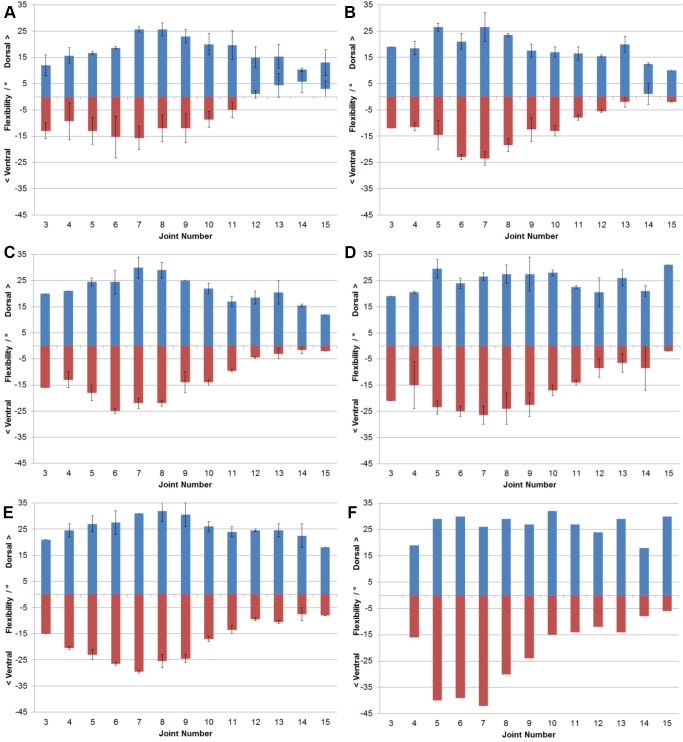
Dorsoventral flexibility of the neck of *Struthio camelus* with tissue removal. Measurements of dorsoventral flexibility of the neck joints of *Struthio camelus* through stages of cumulative tissue removal. (a) All tissues present. (b) Long dorsal muscles removed. (c) Long ventral muscles removed. (d) Long lateral muscles removed. (e) Single-segment muscles removed. (f) Ligamentum elasticum removed. ((a) n = 3; (b-e) n = 2; (f) n = 1).

**Figure 3:**
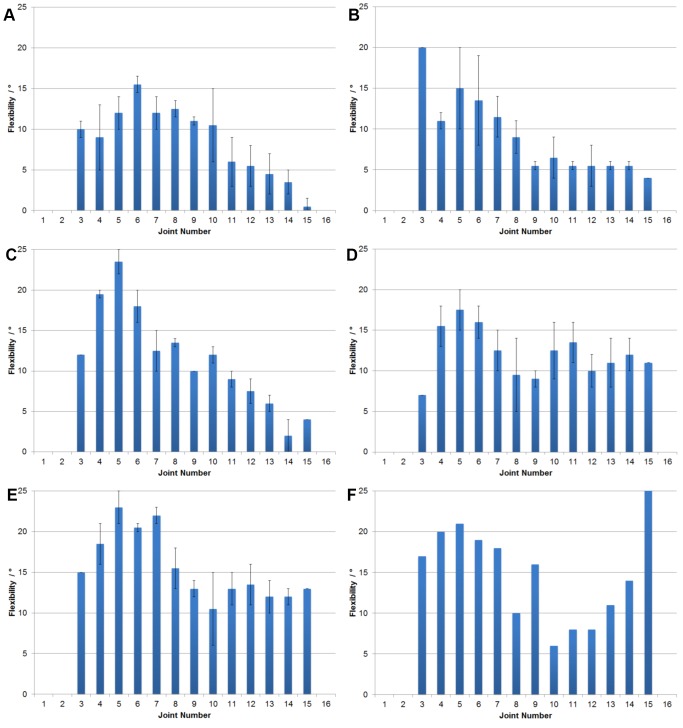
Lateral flexibility of the neck of *Struthio camelus* with tissue removal. Measurements of lateral flexibility of the neck joints of *Struthio camelus* through stages of cumulative tissue removal. (a) All tissues present. (b) Long dorsal muscles removed. (c) Long ventral muscles removed. (d) Long lateral muscles removed. (e) Single-segment muscles removed. (f) Ligamentum elasticum removed. ((a) n = 3; (b-e) n = 2; (f) n = 1).

The maximum dorsoventral flexibility of the ostrich neck after sequential and cumulative removal of muscles was measured (Fig. 2). The flexibility of the ostrich neck with all muscles intact can be divided into three sections (Fig. 2a): between C3–C6, with dorsal extension ranging from 12–19°; C7–C11, with dorsal extension peaking at 25.6° and ranging down to 19.6°; and the posterior section C12–C15, with dorsal extension ranging from 13–15°. Ventral flexion of the neck does not exhibit the same range as dorsal extension, the maximum excursion from 0° being at joint 7 and reaching 15.6°. However, the same tripartite pattern seen in the dorsal flexion can be observed in the ventral flexion. In C12–C15, the vertebrae are unable to flex ventrally below 0°. There is a noticeably larger variation in the ventral flexibilities of the neck in comparison to maximum dorsal excursions. Lateral flexibility follows a similar pattern, with comparatively low values at the anterior end of the neck, increasing to >10° for C5–C10, and then decreasing gradually from C11 to the base of the neck, where there is little flexion (<5°) (Fig. 3a).

Removing the long dorsal muscles of the neck increases flexibility along the whole neck (Fig. 2b). With the removal of these muscles the posterior vertebrae become capable of ventral excursions below the 0° midline, with the exception of joint 14 which is still unable to flex dorsoventrally lower than 1° of dorsal extension. Removing the long dorsal muscles of the neck leads to an increase in lateral flexibility along the neck, allowing for large excursions from 0° from C3–C8, though there is still limited flexibility of a maximum of 6° at the base of the neck (Fig. 3b).

Removing the long ventral muscles of the neck again increases the flexibility both dorsoventrally and mediolaterally (Figs. 2c, 3c); however, this increase is less pronounced than after removal of the dorsal musculature, with the highest increase in flexibility being 4° (C3). The tripartite pattern of dorsoventral flexibility is still apparent, and all vertebrae in the posterior section are capable of ventral flexion.

Removal of the lateral muscles of the neck leads to further increases in dorsoventral flexibility, which are much larger than the increase after removal of the ventral musculature (Fig. 2d). This is especially apparent in ventral flexion, where previously overall ventral flexibility was much lower than that of dorsal flexibility: removal of the lateral musculature leads to comparatively similar flexibility values. However, the ventral flexion capabilities of the posterior section of the neck are still limited, at most reaching 10.5° (C12 and C14). With regards to lateral flexibility, the large differences between the anterior and posterior joints are less apparent after removal of the lateral muscles (Fig. 3d).

The tripartite pattern of flexibility is much less distinct after removal of the single-segment muscles of the neck, leading to another small increase in flexibility (Fig. 2e). Laterally there is a small increase in flexibility, allowing the anterior joints more flexion than those at the base of the neck (Fig. 3e).

Removal of the ligamentum elasticum leads to a large increase in ventral flexibility, especially in joints 5–8 (Fig. 2f). There is no longer any observable pattern in dorsal flexibility.

### Length Measurements

Measurements were taken of the total length of the dorsal side of the neck before and after tissue removal. Prior to tissue removal the average total length of the neck was 76.0+/−4.5 cm (n = 3). After tissue removal, with all vertebral bodies in contact, this length was reduced to 70.1+/−3.75 cm (n = 3). Lengths of the individual centra were also measured after removal of all tissue; whilst still wet, after drying, and after removal of the cartilage caps on each end (Table 2). Drying leads to a mean loss of 0.16+/−0.15 cm in centrum length for each vertebra, whilst removal of the cartilage caps leads to a mean loss of 0.21+/−0.2 cm.

**Table 2:** The effect of cartilage on cervical centra length in *Struthio camelus*.

	Length of vertebral body (cm)		
Vertebra	CartilageWet	CartilageDry	CartilageRemoved
**C3**	4.3	4.0	3.7
**C4**	4.85	4.7	4.5
**C5**	5.55	5.2	5.2
**C6**	5.4	5.3	4.9
**C7**	5.8	5.5	5.35
**C8**	5.9	5.8	5.5
**C9**	6.1	6.0	5.8
**C10**	6.2	6.15	6.1
**C11**	6.5	6.5	6.3
**C12**	6.8	6.7	6.45
**C13**	7.05	7.0	6.7
**C14**	7.1	7.0	6.9
**C15**	7.6	7.3	7.0
**C16**	7.4	7.2	7.0
**Total Length**	86.55	84.35	81.4

Measurements were taken whilst cartilage was wet after boiling off tissue; after 4 days of drying; after removal of the cartilage from the vertebra.

### ONP50

**Figure 4:**
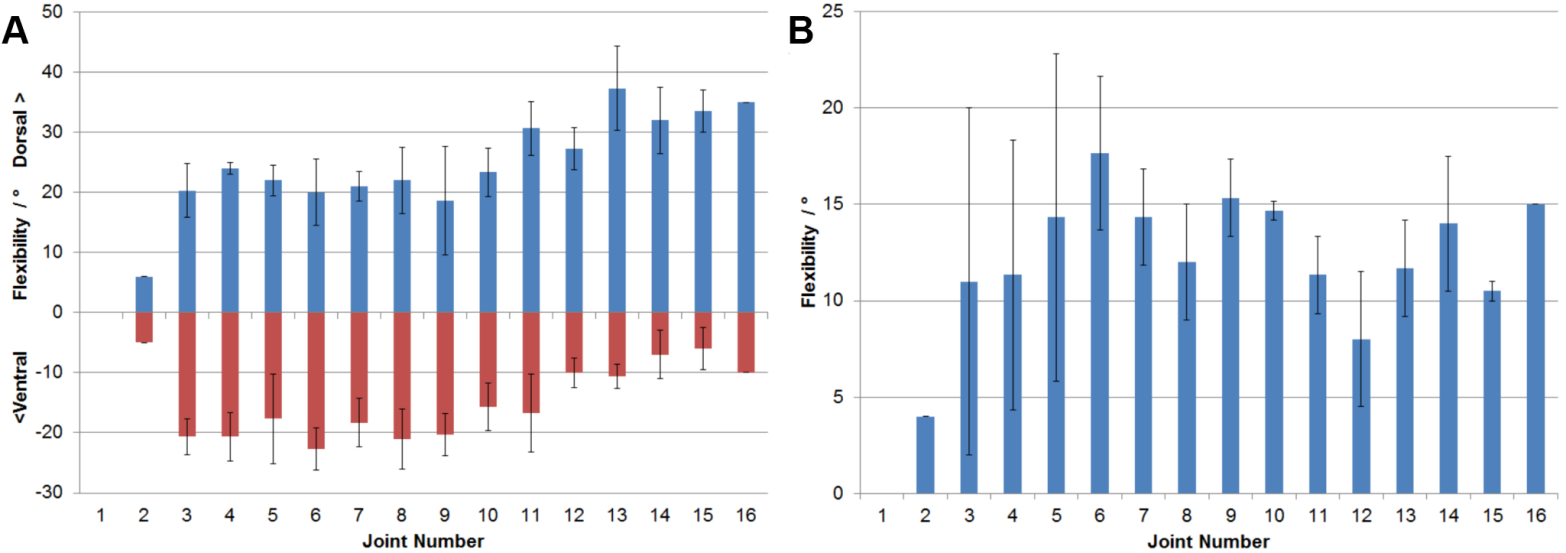
Flexibility allowed by the ONP50 hypothesis in *Struthio camelus.* Measurements of flexibility of the neck skeleton of *Struthio camelus* when limited to a minimum of 50% zygapophyseal overlap, to conform to the ONP50 hypothesis (Stevens & Parrish, 1999). (a) Dorsoventral flexibility. (b) Lateral flexibility. (n = 3).

Measurements of ONP50 in the ostrich neck show that consideration of osteology alone resulted in much higher dorsal and lower ventral flexibilities in comparison with the actual maximum flexibility of the complete ostrich neck. There is no clear pattern of flexibility present, and large variation in the maximum flexibility of specimens studied (Fig. 4a).

ONP50 also allows for a much greater amount of lateral flexibility in comparison to the actual flexibility of the neck. Whilst the pattern of flexibility (high anterior, low posterior) is present, the difference is much less pronounced than shown by the results from the whole neck (Fig. 4b).

### Cartilage

The maximum flexibility of the neck skeleton was measured using ONP50 as a rule for flexibility. Dried cartilage allowed more flexibility than wet cartilage, on average an additional 9.4° (s = 6.1°) of dorsoventral flexibility across the neck (joints 3–15). The flexibility of the neck with the cartilage removed from the vertebrae underwent a large increase in overall dorsoventral flexibility of the neck in comparison with vertebrae with the cartilage present (13.8, s = 9.9°). With both drying and removal, there was large variation in the amount of additional flexibility allowed through a reduction in size or removal of cartilage. This variation occurred in different areas of the neck and both dorsally and ventrally, and no clear pattern was present.

## Discussion

### Flexibility

The general pattern of a division of the neck into three sections with varying flexibility concurs with previous research into the flexibility of avian necks ([43]: pg. 248, fig. 2), where this pattern was observed in other birds both with elongate (*Rhea americana* and *Cygnus olor*) and shorter (*Gallus gallus domesticus* and *Anas platyrhynchos*) necks. The pattern of flexibility with all tissue intact also mirrors that of previous work on the neck flexibility of ostriches ([14]: pg. 707, fig. 7a); however, maximum flexibility in the latter study was judged to be much higher than in our results, with both dorsal and ventral flexibility reaching up to 30° (as opposed to a maximum of 25° dorsally and 15° ventrally as reported herein). The posterior-most vertebrae of the specimens used in our study were also incapable of ventral excursions past the midline of 0°, which is not the case in previous work [14]. However, as the same pattern of flexibility is apparent throughout the length of the neck, it is likely the difference is due to the age of the specimens rather than the sampling method. Whilst this study used juvenile ostriches, adults were used in previous research [14] (see below for further discussion). The inability of the posterior cervicals to flex ventrally beyond the midline at first seems counter-intuitive given the range of motion seen in live ostriches. However, because previous results demonstrate that adult ostriches are capable of these ventral excursions, it is possible that younger (infant or juvenile) ostriches are restricted in their range of motion. Alternatively, the ventral flexion seen in living animals may be due to movements of the anterior dorsal vertebrae, which were not incorporated into this study.

Because the neck musculature controls flexion, it is no surprise that as muscles are removed, maximum flexibility increases. There does not appear to be any group of muscles that specifically affects the total flexibility; though there is a large increase in the maximum dorsal excursions possible in the posterior-most vertebrae after removal of the long, lateral muscles (Fig. 2d), this is likely due to the large amount of tissue that had been removed from those vertebrae (to include the dorsal and ventral muscles). Ventral flexibility is largely limited by the ligamentum elasticum, with extreme excursions possible after the removal of the ligament (Fig. 2f), concurring with previous research [14].

**Figure 5:**
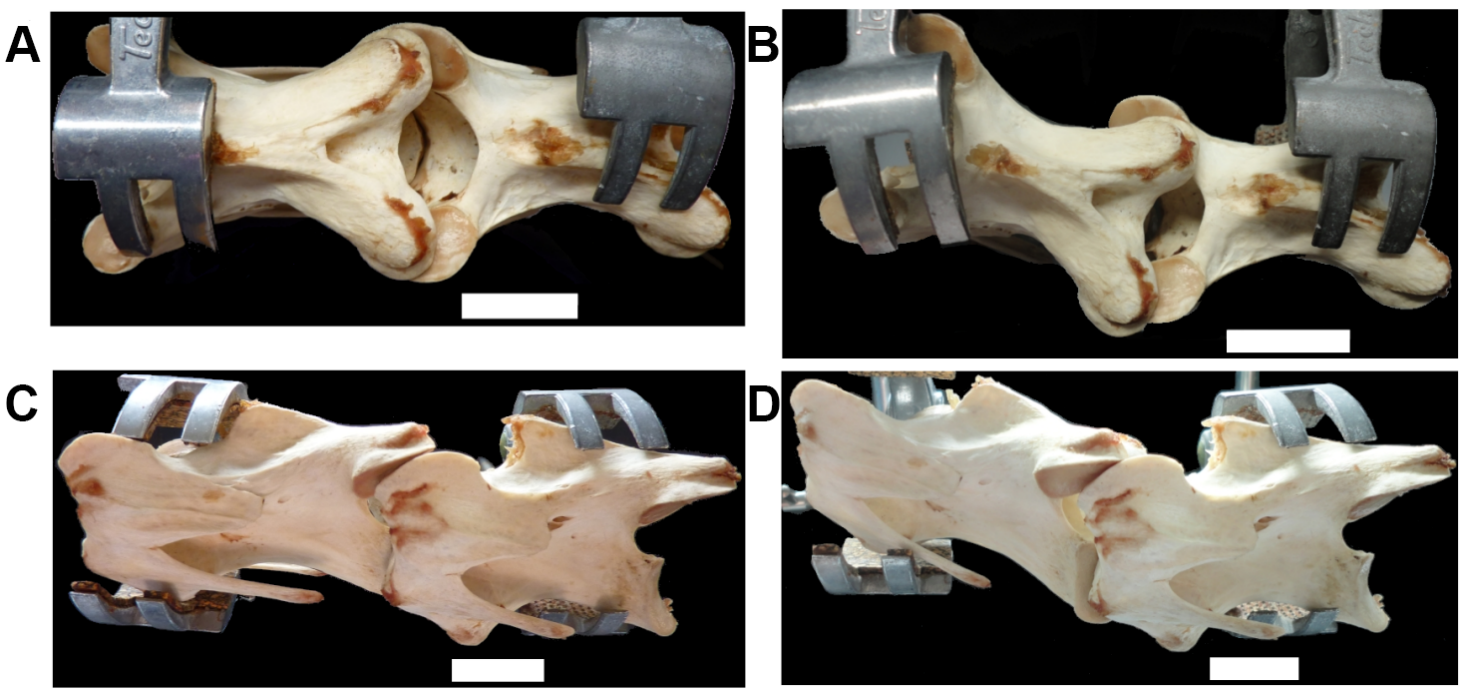
Dorsal flexion as a consequence of laterally flexing the posterior cervical vertebrae of *Struthio camelus*. (a, c) C15 and C16 with no lateral flexion, and flexed ventrally to reach a dorsoventral angle of 0° (see zygapophyseal overlap (a)). (b, d) C15 and C16 flexed laterally, forcing dorsal flexion. Scale bars = 2cm.

The order of tissue removal is unlikely to have had significant effects on the results presented above. Removal of the long dorsal musculature is likely to lead to a larger overall increase in flexibility than removal of the long ventral musculature, regardless of the order of removal, due to the larger amount of tissue present. Moreover, removing lateral or ‘single-segment’ muscles prior to removing either the dorsal or ventral musculature would be unfeasible due to the more superficial location of the longer dorsoventral musculature. For example, it is impossible to remove the mm. intertranversii (a ‘single-segment’ muscle) without removing, or at the very least pulling away, the m. ascendens cervicalis (a long dorsal muscle). Lateral flexibility is affected by tissue removal in the same way, with overall increases in flexibility. However, the pattern observed differs from that reported previously. This study found higher flexibility towards the head and middle of the neck, steadily decreasing towards the base (Fig. 3a), whereas the opposite result has been presented in prior work ([14]: pg. 707, fig. 7b), which documented little flexibility at joint 1, uniform flexibility of around 15° from between C2–C10, and higher flexibility of 20–25° from joints 10–18. This difference is likely due to the dorsal inclination of the posterior-most vertebrae of the neck (Fig. 2a). To measure lateral flexibility, the vertebrae require ventral flexion to become dorsoventrally ‘neutral’ (i.e. 0°). This dorsoventral flexion may have limited lateral flexibility, concurring with prior work: “lateral flexibility is significantly reduced if simultaneously flexed dorsally” ([14]: pg. 707). However, it was observed that when the vertebrae are not flexed ventrally to achieve dorsoventral neutrality, and retain their dorsal inclination, large lateral excursions are possible. When the prezygapophyses of the posterior vertebrae pass further under the postzygapophyses of the anterior vertebrae, the body of the posterior vertebra is inevitably lifted upwards (Fig. 5), leading to dorsal flexion. Inversely, to keep the vertebrae dorsoventrally neutral during larger lateral excursions requires ventral flexion of the anterior vertebrae.

It is probable that these differences are an artefact of the experimental protocols. Whilst both studies measure lateral flexibility with adjacent vertebrae oriented dorsoventrally at 0°, the necks in this study are dorsally inclined – and the neck’s natural ‘neutral’ position is above 0°. The dorsal inclination seen in this study is also potentially due to the use of juvenile specimens, whilst previous work has used adults [14]. As stated above, adults show a much higher degree of flexibility across the whole neck than juveniles. This restriction in flexibility could potentially confer more support for the neck during ontogeny, prior to the ossification of tendons in the cervical column. As recent work has shown that the elongate neck ribs exhibited by sauropods are in fact ossified tendons [46], future work should explore the effect of tendon ossification with age on flexibility of the neck. Furthermore, rather than “lateral flexibility [being] reduced if simultaneously flexed dorsally” [14], it is more likely that lateral flexibility is reduced if the neck is simultaneously flexed dorsoventrally away from its natural inclination.

The amount of musculature surrounding the vertebrae and joints limits the amount of flexibility in the neck. Whilst osteological stops may appear to place absolute limits on a neck skeleton, the amount of musculature around a joint further limits the maximum flexibility *in vivo*. There is relatively little difference in the maximum flexibility of the anterior and posterior joints of a neck with little tissue present (Fig. 2e,f), yet there is a much larger difference in one with all musculature intact, with much lower flexibility allowed in the joints towards the base of the neck. As the volume of musculature is much greater in these posterior vertebrae, compared with that of the middle and anterior sections, it is safe to assume that muscle mass *per se* has a great deal of influence on the flexibility allowed at the base of the neck, and as this varies not only between species but between individuals, emphasis should be placed on the assumed amount of muscle mass when estimating neck flexibility from fossil specimens. The reduction in flexibility is not caused by changes in bone morphology, so caution is clearly necessary when attempting to infer this function on the basis of palaeontological material. With no tissue present, there is no obvious reduction in the excursions possible in the posterior vertebrae.

### ONP50

Positioning the neck in maximal dorsoventral flexion to exhibit 50% overlap of adjacent zygapophyses does not recover the same tripartite pattern of flexibility as seen when the whole neck is manipulated into its maximally flexed posture. Whilst the overall neck flexibility possible is much higher in ONP50, there is relatively less flexibility dorsally in the anterior and middle sections of the neck, with the highest flexibilities allowed in the posterior portion. This is the opposite pattern to that implied by work on intact necks. ONP50 still results in little ventral flexibility at the base of the neck compared to the joints anterior to it, but aside from the small amount of flexibility allowed in the joint between the axis and C3, there is no real differentiation between the anterior and middle sections of the neck. When measuring lateral flexibility there is no clear pattern, whereas with tissues intact there is a higher anterior flexibility, decreasing to very little flexibility at the base of the neck.

These findings undermine the utility of ONP50 as a measure of neck flexibility. Whilst a discrepancy between the values for flexibility under the same pattern would allow compensation for over- or underestimates, no pattern of flexibility between cervical regions is recovered, although this has been found in studies of extant avian taxa (see above).

**Figure 6:**
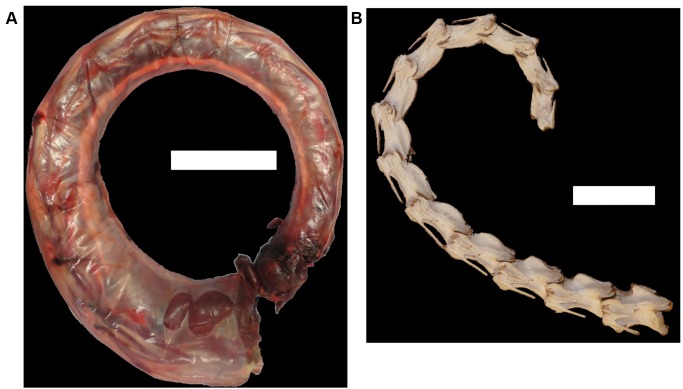
The effect of inter-vertebral space on overall flexibility of the neck of *Struthio camelus*. (a) Neck with all tissues intact in sub-maximal dorsal flexion. (b) The same neck cleaned of all tissue, articulated to match the maximum dorsal flexibility of each joint, with all centra touching. Scale bars = 10 cm.

**Figure 7:**
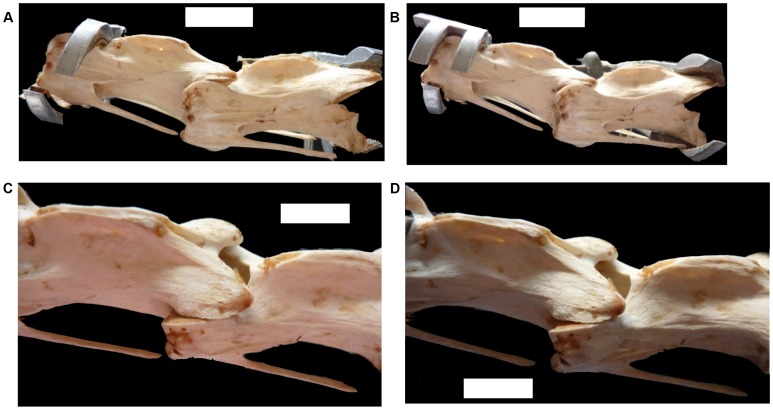
The effect of inter-vertebral space on zygapophyseal overlap in the neck of *Struthio camelus*. (a) Vertebrae C11 and C12 positioned with 20° of dorsal flexion, with no space between centra, with zygapophyseal overlap shown (c). (b) C11 and C12 positioned with 20° of dorsal flexion, with a 0.2 cm gap between centra, and increased overlap of zygapophyses (d). Scale bars = 2 cm.

When comparing vertebral series with wet, dry or absent cartilage, there is a general increase in flexibility with a reduction in centrum length for each joint. This is likely due to an increased amount of room for manoeuvrability between those joints. This has direct consequences for assessments of flexibility based on fossil specimens, whether in ONP50 or through other methods. As the presence of cartilage reduces the amount of flexibility, any attempts to assess flexibility through dry bone alone must be overestimates due to an under-represented total centrum size. However, the length of the neck decreases when all centra (with cartilage intact) are placed in contact with each other. This indicates that the vertebral bodies of the neck are not in constant contact with each other, and there is a varying amount of space allowed between the vertebrae within the synovial capsules. This is best illustrated by comparing the neck in sub-maximum flexibility prior to dissection, and the neck skeleton articulated to fit the maximum flexibility of the neck with all tissue intact, but with all vertebral bodies in contact (Fig. 6). ONP50 does not allow for these deviations, keeping a constant distance between any two vertebrae. As there is this room for manoeuvrability, it is possible that the same amount of flexibility can be obtained with a reduced deviation from neutral zygapophyseal overlap (Fig. 7), allowing increased flexibility with less stress on the synovial capsules.

### Implications for Sauropod Biology

These results show that estimations of neck function based solely on osteological data should be viewed with caution, with serious implications for palaeobiologists. Although the individual muscles comprising the neck musculature of sauropod dinosaurs can be reconstructed on the basis of homologies identified between living taxa (e.g. [47], [48]), palaeontologists lack precise information on the masses and cross-sectional areas of these axial muscles, and thus their roles in neck function are unquantifiable. Moreover, the foregoing comparisons in neck flexion between samples with and without soft tissues imply that previous work ignoring the influence of these tissues on potential flexibility are likely to have seriously overestimated the amount of movement permissible at the inter-vertebral joints. Regardless of whether sauropod necks were held vertically or horizontally, it is possible that they were less flexible, both mediolaterally and dorsoventrally, than has often been assumed. If this was the case, it would suggest that sauropod feeding envelopes, while still large relative to other animals due to the extreme length of the neck [49], were potentially smaller than previously envisioned (e.g. [8], [12], [13]). This in turn would have consequences for niche partitioning and the energetics of these animals, which might have had to forage more actively in order to meet their daily minimum energy budgets.

### Conclusions

- The ostrich neck can be divided into three sections of varying flexibility; a slightly flexible anterior section, a very flexible middle section, and a stiff posterior section.
- The soft tissues of the neck place absolute limits on flexibility, as removal of the muscles leads to higher maximum flexibility. Therefore muscle mass needs to be taken into account in any predictions of flexibility. Osteological reconstructions are insufficient to predict neck flexibility in extinct taxa.
- Zygapophyseal overlaps do not reliably indicate flexibility or the pattern of flexibility across the whole neck. There are variable distances between adjacent vertebral bodies, allowing for increased flexibility in equal amounts of zygapophyseal overlap. Therefore ONP50 is inappropriate as a measure of neck flexibility.
- The amount of cartilage present affects potential flexibility. This requires further work on the role of in neck flexion.
